# Cortical pencil lining on SWI MRI in NBIA and healthy aging

**DOI:** 10.1186/s12883-019-1471-7

**Published:** 2019-10-14

**Authors:** Marlous C. M. van der Weijden, Peter Jan van Laar, Roald A. Lambrechts, Dineke S. Verbeek, Marina A. J. Tijssen

**Affiliations:** 1 Department of Neurology, University Medical Center Groningen, Hanzeplein 1, 9700 RB Groningen, The Netherlands; 2 Department of Genetics, University Medical Center Groningen, Groningen, The Netherlands; 3 Department of Radiology, University Medical Center Groningen, Groningen, The Netherlands; 4 Department of Radiology, Zorggroep Twente, Almelo and Hengelo, The Netherlands; 5 Department of Cell Biology, University Medical Center Groningen, Groningen, The Netherlands

**Keywords:** Iron accumulation, Susceptibility-weighted imaging, Cortical pencil lining, Healthy aging, NBIA

## Abstract

**Background:**

Neurodegeneration with brain iron accumulation (NBIA) is characterized by pathological iron accumulation in the subcortical nuclei and the cortex. As age-related iron accumulation studies in these structures are lacking in healthy aging, we aimed to characterize the dynamics of age-dependent iron accumulation in subcortical nuclei in healthy aging and selected NBIA cases. This is fundamental to understand the natural age-related iron deposition in the healthy brain prior to using this marker as a potential prognostic or diagnostic tool in neurodegenerative disorders.

**Methods:**

Susceptibility-weighted imaging (SWI) scans from 81 healthy volunteers (0-79 years) and four genetically confirmed patients suffering from NBIA (2-14 years) were obtained. We scored the presence or absence of pencil lining of the motor cortex and putamen and analyzed the normalized SWI signal intensity ratio (NSIR) in five subcortical nuclei.

**Results:**

In healthy subjects, an age-dependent increase of pencil lining occurred starting from the second decade of life and was present in all cases at the age of 50. In their first decade, NBIA patients showed no cortical pencil lining, but we did observe putaminal pencil lining at this stage. In healthy subjects, age and NSIR of all nuclei correlated positively and was particularly dynamic in early childhood until young adulthood in the globus pallidus, dentate nucleus and red nucleus, but not in the caudate nucleus and putamen. NBIA patients showed an increased NSIR in the globus pallidus only and not in the other subcortical nuclei compared to age-matched healthy subjects.

**Conclusions:**

Cortical pencil lining is part of healthy aging. This should be considered when assessing this as a potential marker in NBIA diagnosis and prognosis. Putaminal pencil lining has the potential to become a specific marker for some subtypes of NBIA in the first decade of life, as it was only observed in NBIA and not in age-matched healthy subjects. NSIR in the subcortical nuclei during healthy aging was shown to be dynamic, accentuating the importance of having an age-dependent baseline.

## Background

Iron is an essential component for normal brain function including energy production, mitochondrial respiration, protein and DNA synthesis, as well as in the biosynthesis of neurotransmitters and myelin [1–7]. Dysregulation of iron homeostasis can cause neurotoxicity through different mechanisms believed to be responsible for a number of neurodegenerative disorders including neurodegeneration with brain iron accumulation (NBIA) [3, 8, 9]. NBIAs are a genetically and clinically heterogeneous group of movement disorders where iron accumulation occurs in the brain, mostly in the basal ganglia. Up to date, 10 different gene mutations causing NBIA have been identified, but a significant portion of cases remain without a known genetic cause [10, 11].

With current advances in brain imaging techniques, the potential of using different imaging modalities as non-invasive markers for brain iron accumulating diseases has increased, therefore becoming a corner stone for corroborating clinical suspicion. For example, using the T2/T2* MRI sequence, patients with mutations in pantothenate kinase 2 (PANK2) show marked hyperintense signals in the globus pallidus with a hypointense surrounding, known as the “eye of the tiger” sign [12, 13]. This feature is absent in other NBIA subtypes, making its presence a specific sign for PKAN. Additionally, a linear hypointense tracing of the cortical motor area using susceptibility weighted imaging (SWI), referred to as “cortical pencil lining” is suggested to be a potential marker to discriminate neuroferritinopathy patients from other NBIA subtypes (PKAN or PLAN patients) [14].

To assess the discriminatory and/or prognostic potential of such radiologic signs, it is vital to understand the natural age-related iron accumulation in healthy brains. This is particularly relevant given that several post-mortem and in vivo studies have demonstrated an age-related increase in iron deposition in NBIA-linked brain regions of previously healthy subjects [8, 14–18].

For the purposes stated above, we investigated the presence or absence of cortical pencil lining on SWI in 81 healthy subjects ranging from birth to 79 years of age and in 4 genetically confirmed NBIA subjects ranging from 2 to 14 years as a small contrast group. The choice for SWI stems from the present-day use of this sequence in standard clinical care in many neurological evaluations and its sensitivity for detecting iron. In addition, to find potential sensitive discriminative targets for NBIA and to further improve our knowledge of brain iron accumulation in healthy aging, we examined the presence of putaminal lining and the dynamics of age-related SWI signal intensity change in five subcortical nuclei linked to neurodegeneration.

## Methods

### Participants

Brain MRI scans of healthy subjects without known neurodegenerative, psychiatric, cerebrovascular or post-traumatic disease, were included from a research database of the department of Radiology of the University Medical Center Groningen (UMCG), Groningen, the Netherlands. Subjects with the aforementioned diseases were excluded. Written informed consent was obtained from all subjects or their parents. The group included 40 men with a mean age of 39.6 years (range 5-79 years) and 41 women with a mean age of 40 years (range 0-79 years). The demographic data of these 81 healthy subjects is summarized in Table 1.

**Table 1:** Demographic data of healthy study subjects per decade at moment of scanning

Decade	N	M/F	Mean age in years (range)
1	10	5/5	4.4 (0-9)
2	9	3/6	15.1 (10-19)
3	10	6/4	25.1 (22-29)
4	12	8/4	34.5 (30-37)
5	10	5/5	43.7 (41-49)
6	10	5/5	56.3 (51-59)
7	11	4/7	65.0 (61-69)
8	9	4/5	73.9 (70-79)
Total	81	40/41	(0-79)

*N* number of subjects, *M* male, *F* female

Four genetically diagnosed NBIA patients were also included, three with a mutation in the WDR45 gene (BPAN) and one case with a mutation in the PANK2 gene (PKAN) (see Additional file 1: Table S1 for further details). The BPAN patients were diagnosed at 6, 7 and 14 years of age and the PKAN patient at 2 years of age. The corresponding SWI scans were made at the time of diagnosis.

### MRI acquisition

Imaging was performed using a 1.5-Tesla whole body system (Magnetom Aera, Siemens, Erlangen, Germany). The SWI sequence was a 3D velocity compensated gradient echo sequence. Magnitude and Phase images were obtained in the axial plane. The Phase images were high pass filtered to remove unwanted artefacts. The magnitude image was combined with the Phase image to create an enhanced contrast magnitude susceptibility image. The parameters of the SWI sequence were Repetition Time (TR): 49 ms, Echo Time (TE): 40 ms, Flip angle: 15°, Field of View read: 230 mm, slices per slab: 80, slice thickness: 1.6 mm, voxel size: 0.7 × 0.7 × 1.6 mm, phase encoding direction: R> > L and transversal orientation, bandwidth: 80 Hz/Px with flow compensation, averages: 1, and total scan time: 6:15 min.

### Image analysis

All SWI scans were blindly analyzed by a senior neuroradiologist (PL). The SWI sequence can show a fine band of low signal following the contour of the motor strip of the cerebral cortex and in the periphery of the putamen. This appears as a thin line of low signal looking as if traced with a black pencil [14]. This pencil lining in the motor strip of the cerebral cortex was scored with a system based on previous methods described in the literature as present or absent [14].

To assess SWI signal intensity in the caudate nucleus, putamen, globus pallidus, red nucleus and dentate nucleus, we used a region-of-interest (ROI) method similar to Meijer et al. (2015), with the exception of using a grading system for the signal intensity values.[19] In short, the ROI method was used to evaluate the SWI sequences on a PACS workstation (Carestream). A 5 mm^2^ circular ROI was placed bilaterally in the caudate nucleus, putamen, globus pallidus, red nucleus and dentate nucleus on a single slice. Given that no significant differences in intensities between the normalized SWI scan values of the ROIs of both hemispheres were observed (paired-samples t-tests *p* ≥ 0.8), the obtained intensities for the nuclei of both hemispheres were averaged. The signal intensity of the CSF was measured by ROI placement in the lateral ventricle. To limit the potential non-uniformity of the CSF signal, the signal intensity of two measurements bilaterally in the lateral ventricle were averaged. Additionally, the ROIs were placed avoiding vessels, flow voids, the choroid plexus and were not including the edges of the structures. The ROI method was performed by a senior neuroradiologist (PL) blinded to the clinical symptoms and diagnosis. See Additional file 1: Figure S1 for an example placement of an ROI bilateral in the lateral ventricle and the left caudate nucleus.

To correct for inconsistencies in the reference standard, we calculated the ratio of the SWI signal intensity in the cerebrospinal fluid (CSF) of the lateral ventricle (LV) to the signal intensity of the different nuclei (normalized signal intensity ratio (NSIR) = SWI signal intensity of the LV / SWI signal intensity of the subcortical nucleus).

### Statistical analysis

All statistical analyses were performed with SPSS (version 23, IBM, USA). Prior to analyzing the effect of age on brain iron deposition, potential confounding factors were tested by assessing possible imbalances within and between decades of life. In addition, distributions and assumptions within the group of healthy subjects were checked and because of the non-normality of the data, non-parametric testing was used to compare for group differences and gender effects (Mann-Whitney U test). Given that the healthy subjects showed an equal distribution of gender, mean age, and number of subjects between and within each decade of life, we did not include these factors in the study analyses. Analysis of Variance (ANOVA) was performed to evaluate potential group differences. To correct for multiple comparisons between the five ROIs, a Bonferroni corrected *p*-value ≤0.01 (0.05/5) was considered significant for omnibus F-tests. To evaluate the correlation between age and brain iron content, a Spearman’s rank correlation coefficient was calculated for the different ROIs. Figures were made in R (version 3.0.0, R Foundation for Statistical Computing, Vienna, Austria). Since no equal distributions between NBIA patients and healthy subjects was present due to the small number of patients, no statistical analysis was performed between the two groups.

## Results

### Cortical pencil lining

**Fig. 1:**
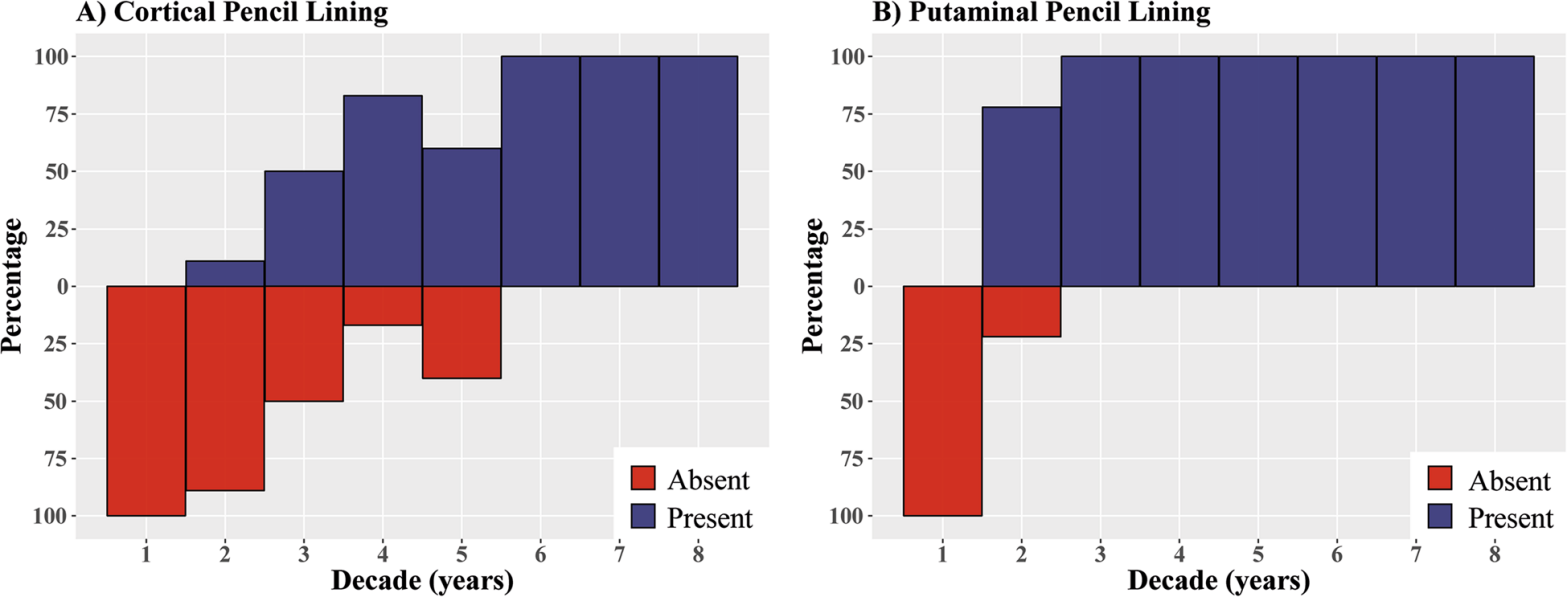
Age-related iron deposition lining the motor cortex and putamen in 81 healthy subjects. The graph shows the percentage of healthy subjects (Y-axis) per decade of life (X-axis) that exhibit hypointense signals on SWI in (**a**) the motor cortex and (**b**) the putamen

The qualitative SWI scan analysis revealed that the presence of cortical pencil lining was positively correlated with age. More specifically, up to 50% of the healthy subjects in their third decade of life showed cortical pencil lining as well as all subjects older than 50 years of age (Fig. 1 and Additional file 1: Figure S2). In Additional file 1: Figure S2 differences in the presence or absence of cortical pencil lining in healthy subjects age 35 are shown. No cortical pencil lining was observed in healthy subjects in their first decade of life.

Notably, no cortical pencil lining was observed in four NBIA patients (range 2-14 years), suggesting that cortical pencil lining is not temporally linked to the pathological iron accumulation seen in the globus pallidus of these young PKAN and BPAN cases.

### Putaminal pencil lining

**Fig. 2:**
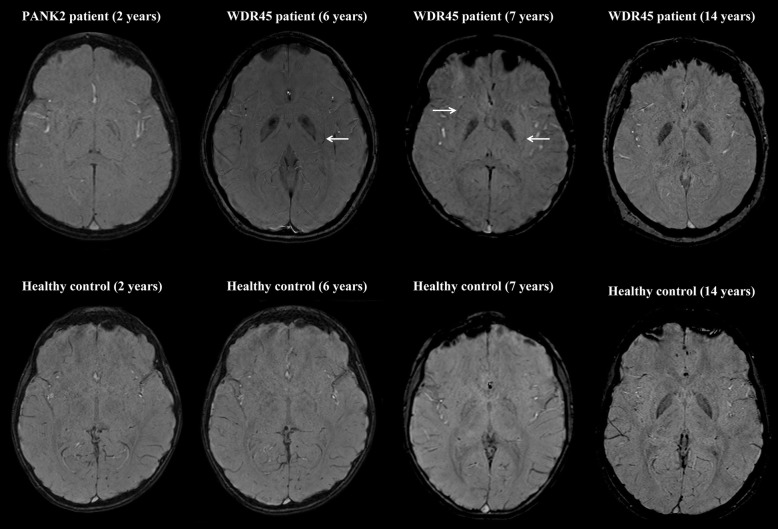
Presence or absence of putaminal pencil lining. Representative SWI images of NBIA patients (WDR45 and PANK2 mutation carriers) and age-matched healthy subjects (2, 6, 7 and 14 years old) showing the absence or presence of putaminal pencil lining

The presence of putaminal pencil lining was also positively correlated with age. All subjects older than 20 years of age exhibited putaminal pencil lining (Fig. 1). No putaminal pencil lining was observed in healthy subjects in their first decade of life. Putaminal pencil lining was observed in two BPAN cases who were diagnosed in their first decade of life, but was absent in the 2-year-old PKAN and 14-year-old BPAN patients (Fig. 2).

### Subcortical normalized signal intensity ratio (NSIR)

**Fig. 3:**
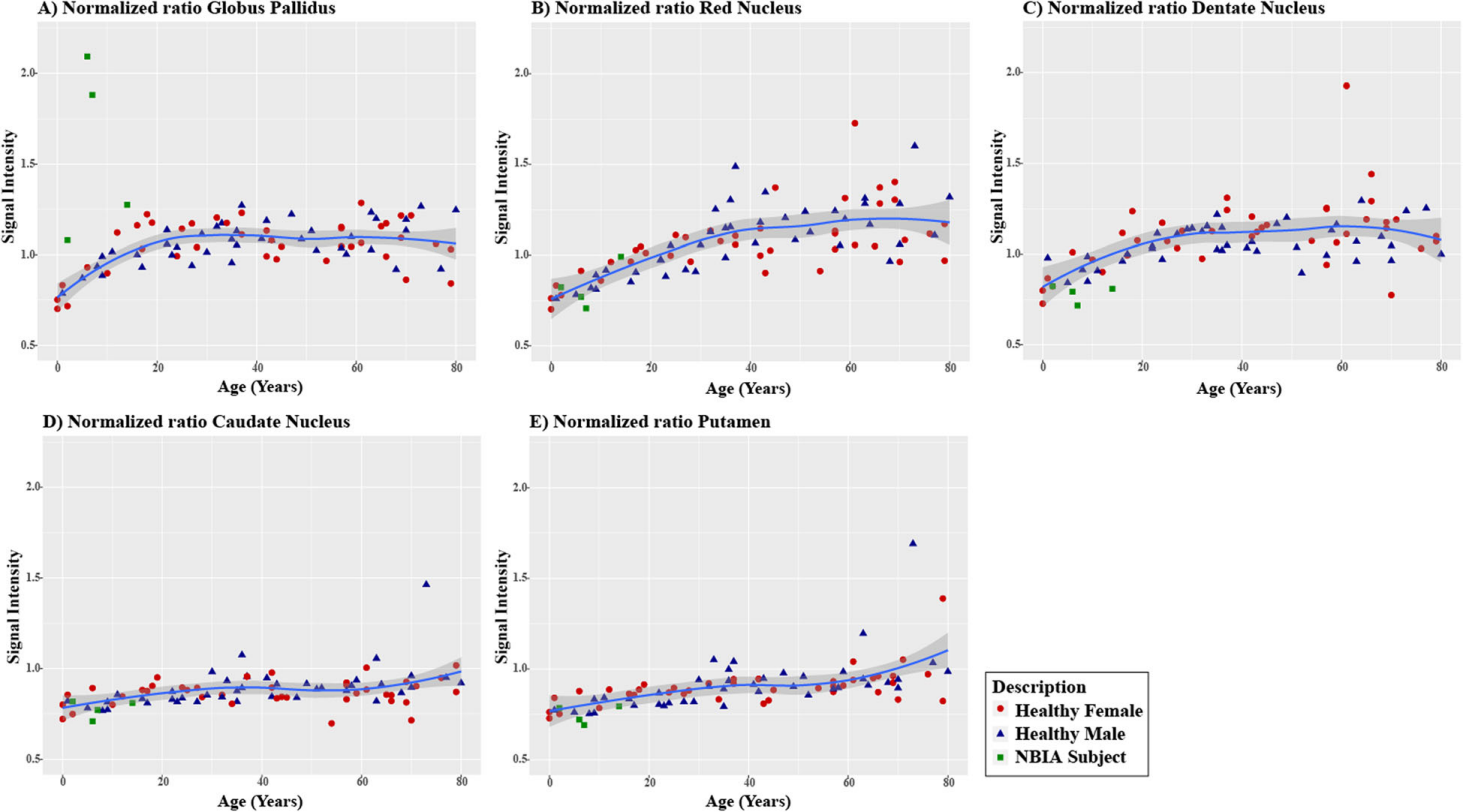
Age-related regional SWI signal intensity. Normalized signal intensity ratio (NSIR) (ratio between the signal intensity of the lateral ventricle and a region of interest (ROI); right and left hemisphere values are averaged) and the signal intensity of the ROI in five subcortical nuclei: globus pallidus (**a**), red nucleus (**b**), dentate nucleus (**c**), caudate nucleus (**d**), and putamen (**e**) over time. Red rounds, blue triangles and green squares denote healthy females, healthy males and NBIA subjects, respectively. The thick continuous line is the smoothened conditional mean of healthy subjects. The shade around the line represents the simultaneous prediction bounds of the 95% confidence intervals for the mean. X-axis: age in years; Y-axis: NSIR

To further study the natural dynamics of iron accumulation in the brain during healthy aging, we investigated the normalized signal intensity ratio (NSIR) of SWI scans of the caudate nucleus, putamen, globus pallidus, red nucleus and dentate nucleus in our cohort of healthy subjects over different decades. We observed significant positive correlations between age and the NSIR of all subcortical nuclei (Fig. 3). The strongest correlations were detected for the red nucleus and putamen (both R^2^ of 0.67, *p* < 0.001; Fig. 3b and e), whereas weaker but still significant correlations were observed for the caudate nucleus, globus pallidus, and dentate nucleus (*R*^2^ of 0.44, 0.35, and 0.47, respectively; all *p* < 0.001). The globus pallidus showed a marked significantly increased NSIR during the first decade of life (*p* < 0.01) followed by a plateau phase starting around 20 years of age (Fig. 3a and Table 2). This suggests that iron accumulation occurs relatively fast early in life in the globus pallidus. The NSIR of the red nucleus and dentate nucleus significantly increased up to 40 years of age (*p* < 0.01), and was then followed by a plateau phase, suggesting that iron accumulated more gradually over time in these subcortical nuclei. Additionally, the caudate nucleus and putamen showed a more linear increase of NSIR, reflecting a quite modest iron accumulation over time (Fig. 3 and Table 2). The increase in NSIR was only significantly different between the first and eighth decade of life in the putamen (*p* < 0.01), whereas significantly increased NSIR were observed between the first three and the eighth decade of life in the caudate nucleus (*p* < 0.01).

**Table 2:**
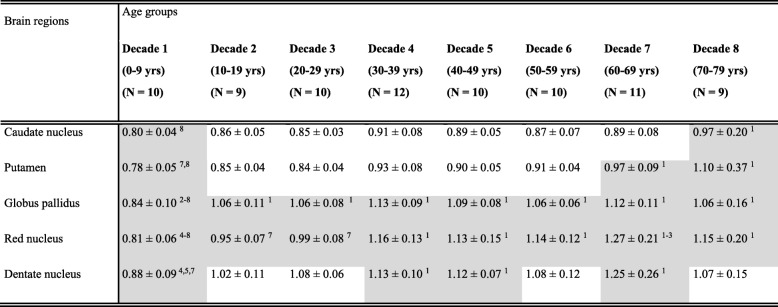
Quantitative age-related iron accumulation indicated by NSIR signal intensities per nucleus

Shaded boxes: significant Bonferroni corrected differences (*p* < 0.01). Numbers denoted in superscript ^x^ = significant difference compared to decade ^x^. Data is shown as mean ± SD

The four NBIA patients showed an increased NSIR in the globus pallidus compared to age-matched healthy subjects that was not observed for the other subcortical nuclei. In addition, the dynamics of the NSIR in the other subcortical nuclei of the NBIA cases showed a similar pattern as seen in healthy subjects (Fig. 3). Although no statistical analysis could be performed due to the small number of NBIA cases, our SWI scan data points to pathological iron accumulation in the globus pallidus as a hallmark of NBIA.

## Discussion

This study, where iron imaging data from the very early decades of life are reported, showed that cortical pencil lining is potentially less specific as a general marker for NBIA. Instead, this sign also appears to be a part of healthy aging as it is encountered in the majority of healthy individuals older than 30 years of age. On the other hand, putaminal pencil lining in the first decade of life was seen in specific NBIA cases only and not in age-matched healthy individuals suggesting its potential as a specific marker of pathological iron accumulation in specific subtypes of young NBIA patients. Finally, this work characterized NSIR in subcortical nuclei of the brain during healthy aging, where a positive correlation between age and NSIR in all subcortical nuclei was shown.

None of our NBIA patients, including BPAN and PKAN, showed cortical pencil lining in their first or second decade of life. The presence of cortical pencil lining was seen in three neuroferritinopathy cases (37, 54 and 79 years of age) as reported in the work of Batla et al. [14]. Although we were not able to include neuroferritinopathy cases in our study, the findings in our study suggest that the presence of cortical pencil lining in these neuroferritinopathy cases might at least partly be due to age, as 80% of the healthy cases in our cohort showed cortical pencil lining between 30 and 40 years and more than 50% between 20 and 30 years of age. Additionally, this might also be explained by a more diffuse iron accumulation throughout the brain in neuroferritinopathy cases, whereas the iron accumulation in BPAN and PKAN cases is more isolated to the basal ganglia.

The presence or absence of cortical pencil lining in patients older than 20 years of age can therefore not be used as a specific marker. Alternatively, we speculate that quantification of the different pencil linings may discriminate between NBIA subtypes and between NBIA patients and healthy subjects. Such quantification has been demonstrated to be useful in the diagnosis of amyotrophic lateral sclerosis (ALS) and primary lateral sclerosis (PLS) [20, 21]. It should be noted that current approaches for diagnosing NBIA subtypes are based on a combination of clinical characteristics, genetic testing and MRI findings. Nevertheless, in 30% of the cases no genetic confirmation is found and the diagnosis can only be made on MRI findings coupled with clinical suspicion [22]. Furthermore, because of the broad range of possible clinical features present in NBIA, MRI findings often provide the first strong clue for the diagnosis. This further accentuates the importance of this kind of research providing a normal baseline for a frequently used MRI sequence.

To our knowledge, this is the largest study on iron deposition in the brain using SWI focusing on healthy subjects that also includes individuals in their first decade of life. This period of life is of particular interest given the age of onset of the classic disease types of NBIA. This is further substantiated by the identification of putaminal pencil lining exclusively in young NBIA cases but not in age-matched healthy individuals. This suggests that the occurrence of putaminal pencil lining in the first decade of life may be a potential marker in specific subtypes of NBIA, as none of the healthy subjects in their first decade of life had any signs of putaminal pencil lining.

In the early period of life we observed dynamic iron accumulation in the globus pallidus, red nucleus, and dentate nucleus. The non-linear pattern of SWI signal intensity change reflecting iron accumulation in the globus pallidus was reported before [18]. However, the exponential increase of iron accumulation in the red nucleus and dentate nucleus has, to our knowledge, not been described before. In contrast, the caudate nucleus and putamen showed a more gradual increase which is in line with previously published work using T2* and quantitative susceptibility mapping MRI [23–27]. It appears that early onset neurodegenerative disorders associated with aberrant iron homeostasis such as subtypes of NBIA and Friedreich’s ataxia preferentially show increased iron accumulation in nuclei where there is exponential increase in iron content early on in life, such as the globus pallidus and dentate nucleus [28, 29]. When compared to acquired, late onset, neurodegenerative disorders such as Parkinson’s disease, multiple system atrophy and Alzheimer’s disease we mostly find preferential iron accumulation in different nuclei, such as the caudate nucleus and putamen [19, 30, 31]. Speculatively, this may point to a primary versus secondary effect of pathological brain iron accumulation, i.e. accelerated accumulation leading to pathology in NBIA cases and secondary excess iron accumulation due to neurodegeneration leading to further damage in acquired neurodegenerative disorders.

Some limitations should be noted in this study. First, the sample size of subjects per decade is relatively small and only a few cases of NBIA are examined as a contrast group. Future studies including higher subjects are advisable to confirm the validity and normal ranges of these results. Second, although SWI was used, which is a well-established surrogate marker for in vivo iron quantity [32], the signal intensity may be affected by other components such as calcium and lipids. However, the use of a standardized scan sequence widely used in the clinic, as well as using the same MRI scanner are strengths of this study making direct comparisons between subjects possible. The results presented here are derived from a 1.5-Tesla field strength scanner and can be used as a reference for this field strength. Nevertheless, it is important to validate these results on higher scanner field strengths with higher signal-to-noise ratios such as 3-Tesla before articulating definitive recommendations on using this method as a biomarker.

## Conclusions

In this study, we showed that cortical pencil lining may not be a biomarker for NBIA in general. In contrast, putaminal pencil lining could potentially be a useful marker to discriminate between specific subtypes of young NBIA patients and healthy subjects. The “natural” level of age-related iron content in the globus pallidus should be taken into consideration when interpreting SWI scans of NBIA cases or individuals with other neurodegenerative disorders with iron accumulation. Finally, we provide a normal baseline for brain iron accumulation using NSIR during eight decades of healthy aging and suggest to further explore iron levels of the red and dentate nuclei to gain further insights in the significance of their dynamic iron accumulation.

## Additional file

**Additional file 1: Table S1.** NBIA patient demographics. **Figure S1.** SWI with the positioning of the circular region-of-interest. **Figure S2.** Presence or absence of cortical pencil lining.

## Data Availability

The datasets used and/or analysed during the current study are available from the corresponding author on reasonable request.

## Abbreviations

ALS: Amyotrophic lateral sclerosis
ANOVA: Analysis of variance
BPAN: Beta-propeller protein-associated neurodegeneration
CSF: Cerebrospinal fluid
LV: Lateral ventricle
NBIA: Neurodegeneration with brain iron accumulation
NSIR: Normalized SWI signal intensity ratio
PKAN: Pantothenate kinase associated neurodegeneration
PLA: primary lateral sclerosis
PLAN: PLA2G6-Associated Neurodegeneration
ROI: Region-of-interest
SWI: Susceptibility-weighted imaging
TE: Echo time
TR: Repetition time
