# Supplementary material for: Cortical pencil lining on SWI MRI in NBIA and healthy aging
Source: BMC Neurol. 2019 Oct 14;19:233. doi: 10.1186/s12883-019-1471-7 (PMC6790995; doi:10.1186/s12883-019-1471-7)
Supplement: Supplementary file 1 — Additional file 1: Table S1. NBIA patient demographics. Figure S1. SWI with the positioning of the circular region-of-interest. Figure S2. Presence or absence of cortical pencil lining. [file 12883_2019_1471_MOESM1_ESM.docx]

Cortical pencil lining on SWI MRI in NBIA and healthy aging

Marlous C.M. van der Weijden^1,2,*^, Peter Jan van Laar^3,4^, Roald A. Lambrechts^1,5^, Dineke S. Verbeek^2,#^, Marina A.J. Tijssen^1,#^

^1^ Department of Neurology, University Medical Center Groningen, Groningen, The Netherlands

^2^ Department of Genetics, University Medical Center Groningen, Groningen, The Netherlands

^3^ Department of Radiology, University Medical Center Groningen, Groningen, The Netherlands

^4^ Department of Radiology, Zorggroep Twente, Almelo and Hengelo, The Netherlands

^5^ Department of Cell Biology, University Medical Center Groningen, Groningen, The Netherlands

# Shared last authors

* Corresponding author: Marlous C.M. van der Weijden, Department of Neurology, University Medical Center Groningen, Hanzeplein 1, 9700 RB, Groningen, The Netherlands.

Tel: +3150 3612401. Email: [m.c.m.van.der.weijden@umcg.nl](mailto:m.c.m.van.der.weijden@umcg.nl)

Additional file 1

Additional file 1: Table S1 NBIA patient demographics

| NBIA | Mutation | Gender | Age at scanning | Age of onset (Years) | Symptoms |
| --- | --- | --- | --- | --- | --- |
| BPAN | WDR45 (C.28.1 C>GP. TY RE 127*) | F | 6 | 2 | Developmental delay, verbal delay, reduced IQ. |
| BPAN | WDR45 (c.250G>T, p.(Asp84Tyr) | F | 7 | 1 | Global developmental delay, verbal delay, focal epileptic seizures, low muscle tone in extremities. |
| BPAN | WDR45 (c.698G>A; p.Arg233His) | M | 14 | 4 | Moderate developmental delay, focal epileptic seizures, distal myoclonus in the hands, behavioral problems. |
| PKAN | Compound heterozygote: PANK (c.1317del, p(Arg440fs)) + del exon 1-6 | M | 2 | 2 | Developmental delay, dysarthria, posturing of left hand and foot, abnormal gait. |

**
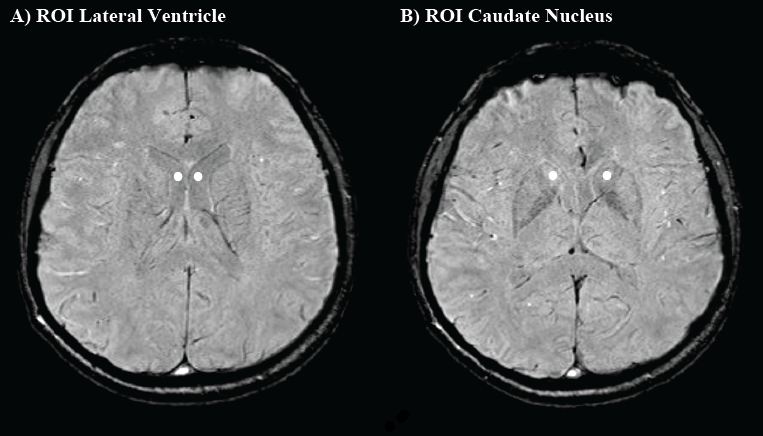
**

Additional file 1**: Figure S1: SWI with the positioning of the circular region-of-interest in the cerebrospinal fluid of the lateral ventricle (A) and in one of the brain structures of interest, the caudate nucleus (B).**

**
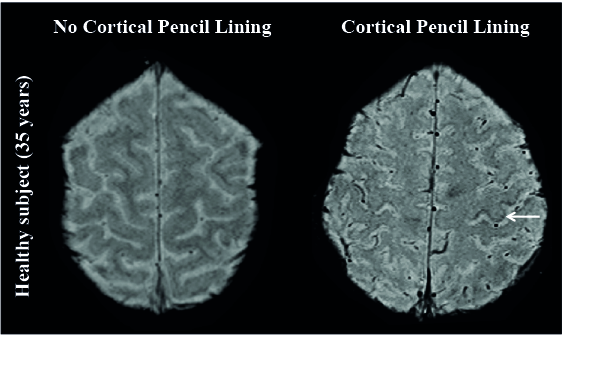
**

Additional file 1: **Figure S2: Presence or absence of cortical pencil lining. Representative SWI images of healthy subjects (35 years old) showing the absence or presence of cortical pencil lining. Cortical pencil lining indicated with an arrow.**
